# Changing patient preferences toward better trial recruitment: an ethical analysis

**DOI:** 10.1186/s13063-023-07258-4

**Published:** 2023-03-28

**Authors:** Pepijn Al, Spencer Hey, Charles Weijer, Katie Gillies, Nicola McCleary, Mei-Lin Yee, Juliette Inglis, Justin Presseau, Jamie Brehaut

**Affiliations:** 1 Rotman Institute of Philosophy, Western University, London, ON Canada; 2 Prism Analytic Technologies, Cambridge, MA USA; 3 Departments of Medicine, Epidemiology & Biostatistics, and Philosophy, Western University, London, ON Canada; 4 Health Services Research Unit, University of Aberdeen, Aberdeen, UK; 5 Centre for Implementation Research, Clinical Epidemiology Program, Ottawa Hospital Research Institute, Ottawa, ON Canada; 6 School of Epidemiology and Public Health, University of Ottawa, Ottawa, ON Canada; 7 Patient partner, Montreal, QC Canada; 8 Patient partner, Edmonton, AB Canada

**Keywords:** Randomized controlled trial, Recruitment, Behavior change techniques, Research ethics, Autonomy, Manipulation

## Abstract

While randomized controlled trials are essential to health research, many of these trials fail to recruit enough participants. Approaching recruitment through the lens of behavioral science can help trialists to understand influences on the decision to participate and use them to increase recruitment. Although this approach is promising, the use of behavioral influences during recruitment is in tension with the ethical principle of respect for persons, as at least some of these influences could be used to manipulate potential participants. In this paper, we examine this tension by discussing two types of behavioral influences: one example involves physician recommendations, and the other involves framing of information to exploit cognitive biases. We argue that despite the apparent tension with ethical principles, influencing trial participants through behavior change strategies can be ethically acceptable. However, we argue that trialists have a positive obligation to analyze their recruitment strategies for behavioral influences and disclose these upfront to the research ethics committee. But we also acknowledge that since neither trialists nor ethics committees are presently well equipped to perform these analyses, additional resources and guidance are needed. We close by outlining a path toward the development of such guidance.

**Supplementary Information:**

The online version contains supplementary material available at 10.1186/s13063-023-07258-4.

## Background

While randomized controlled trials are essential to health research, many trials fail to recruit sufficient numbers of participants [1–7]. For example, between 2004 and 2016, 56 of 151 trials published in the UK’s National Institute for Health Research Health Technology Assessment journal reported failing to meet 80% of their original recruitment target [4]. Such under-enrollment leads to underpowered or biased studies, expensive recruitment period extensions, or, in some cases, trial termination [1, 2, 5–7]. Indeed, a recent analysis of the ClinicalTrials.gov database from 2015 to 2017 found that 39% (1483/3785) of all terminated clinical trials were the result of recruitment issues (see Additional file 1). In addition to being a financial and operational issue, this pattern of failed recruitment is an important ethical issue for health research in general, since each failed trial exposes participants to burdens with little or no prospect of future benefit [1, 4, 6, 7].

Many attempts to address these recruitment challenges have focused on improving or streamlining elements of the informed consent documentation, given that consent documents are a key means by which trial participants learn about the trial [8]. However, focusing on the documentation used during the initial participant consent discussion often does not take into account that research participation is a process that extends beyond that initial decision [9, 10]. Other aspects of the total trial experience (e.g., interactions with the research staff, the size of the commitments of participation, and being informed of study progress and trial results) also may contribute to the overall recruitment and retention [11]. Therefore, the focus of work aiming to improve trial recruitment processes has shifted toward examining how participants’ understanding, decision-making, and trial experience can be supported during and after the consent process [10, 12–14], including how recruiters interact with participants [15].

To better understand and target the drivers and barriers in trial recruitment, some have started to view trial participation through the lens of behavior change theory [15–21]. This approach views trial participation as a behavior, one affected by the same wide range of factors known to affect other behaviors (such as social influences, environmental context and resources, and beliefs about capabilities) [16–19], and which has been the subject of considerable research over the last 50 years [22, 23]. It also emphasizes that recruitment and retention may involve multiple different component behaviors by different stakeholders (e.g., health care provider requests, information disclosure by recruiters, participant consent, participant adherence to protocols, data provision). Behavioral scientists have developed detailed frameworks for identifying and explaining the wide range of strategies that influence human behavior (e.g., Michie and colleagues’ taxonomy of 93 different behavior change techniques) [22, 23]. These strategies can be combined in different ways to develop interventions tailored to and optimized for the situation. While some behavioral influence strategies are already routinely utilized in the trial recruitment process (e.g., providing and shaping people’s knowledge of trials, structuring environments to support trial participation), a more theoretically informed and explicit marshaling of these strategies represents a promising path toward improving recruitment success.

However, at least some behavioral influences could be used to manipulate potential participants, and this prima facie brings them into tension with the ethical principle of respect for persons [24, 25]. The rich literature on undue influence—when potential participants are offered outsized, typically monetary, rewards for trial participation—has covered some of this ground [26, 27]. But there remain underexplored questions about other strategies of behavioral influence that may be used to promote recruitment. For example, May trusted health professionals disclose information about a trial to their patients? May they encourage participation? What are the acceptable boundaries around framing information to promote participation? and May trialists leverage cognitive biases or appeal to the emotions of potential participants during the recruitment process?

In what follows, we discuss two examples of behavioral influences that raise just these questions: one example involves physician recommendations, and the other involves framing of information to exploit cognitive biases. We argue that despite the apparent tension with ethical principles, influencing trial participants through behavior change strategies can be ethically acceptable. However, we argue that trialists have a positive obligation to analyze their recruitment strategies for behavioral influences and disclose these upfront to the research ethics committee. But we also acknowledge that since neither trialists nor ethics committees are presently well-equipped to perform these analyses, additional resources and guidance are needed. We close by outlining a path toward the development of such guidance.

## Is it ethical to leverage trust relationships?

One influence on people’s decision to participate in research is the social influence of trust relationships. Family and friends, for example, can have a strong influence on the decision to participate [28, 29]. The trust participants have in researchers or institutions (e.g., a university or hospital) may also be a reason to participate [30–32], while distrust in pharmaceutical companies has been connected to a decreased willingness to participate in their trials [33].

Another important trust relationship that could be leveraged in recruitment is the trust of patients in their physicians. Patients trust their physicians to recommend treatments or procedures that are in their interests [30]. Such trust is reasonable, insofar as physicians have a duty of care to their patients and are obligated to act and advise so as to promote their medical interests [34].

Given this trust, it is perhaps not surprising to find that physicians can exert a powerful influence on patient decisions to participate in trials. Kinney and colleagues, for instance, surveyed women who attended an informational meeting on a breast cancer prevention [35]. They found that women who discussed trial participation with their physician and were advised to participate were 13 times more likely to do so compared to those who discussed participation but were advised not to enroll.

Both explicit and implicit recommendations (e.g., referrals to trials) are regularly used to encourage participation in trials, and as such, physician recommendation is a potentially important behavioral influence. An example of its use can be found in a study by Embi and colleagues, who developed an electronic notification system that alerted physicians when their patients were eligible for enrollment in a trial [36]. They found that this system increased the number of referrals by physicians 10 times and doubled the number of patients ultimately recruited compared to a no-alert control.

Is it ethical to leverage the trust relationship between physicians and their patients to promote trial participation? Since healthcare is built upon a foundation of patient trust in the integrity of physician judgment, reckless leveraging of that trust may pose a significant social risk. Does such leverage represent too great a risk to the integrity of the research and healthcare enterprises?

For the sake of the present argument, we set aside concerns about the therapeutic misconception (i.e., the incorrect assumption held by research participants that the primary purpose of research is to provide therapeutic benefit) [37, 38]. There is an extensive literature on the subject, and we agree that appreciating the distinction between research and routine care is critical to a valid informed consent. But even if a patient understands the difference between research and care, we believe that there are other ethical conditions that are important to consider.

First, we should acknowledge the substantial power differential between physicians and their patients. Patients are dependent on their physicians for their expertise, advice, and care. This power asymmetry can undermine the voluntariness of the patient’s decision to participate in research [39, 40]. For example, patients may feel obliged to participate if asked to do so by their physician and may fear that a refusal could have negative consequences for their health or for their relationship with the physician.

Next, we should observe the potential for conflict of interest [39]. For example, if physicians are recommending participation in studies run by their colleagues or institutions of employment, their judgment about the appropriateness of trial participation could be (or could perceive to be) biased. Similarly, if the physician (or anyone on their team or staff) is financially compensated for referring patients to a study, their judgment may be biased. To be clear, such biases do not require physicians to be acting with any kind of malintent or negligence. Nor is a positive recommendation to participate always contrary to the interests of the patient. When a patient has a rare disease, for example, participation in a trial can provide access to a desirable therapeutic option. The point is simply that under these conditions, the physician’s fiduciary duty to promote the patient’s interest will be, or may be seen as, affected by other personal, financial, or professional interests, and this may undermine trust.

Given these four factors—the power of physician recommendation, the centrality of trust to healthcare and research, the vulnerability of patients, and the potential for conflicts of interest—we believe that protections are required. Although a comprehensive inventory of the protections is beyond the scope of this paper, we believe that at least one condition is clear: conflicts of interest should be avoided where reasonably possible. Financial incentives that pay physicians a “finder’s fee” to recruit patients are particularly worrisome and, consistent with international ethical guidance, should be avoided [40]. Other financial rewards (e.g., compensating physicians for their time) might be permissible, but do require ethical evaluation to avoid undesirable, unintended consequences. When avoiding conflicts of interest, special caution should be taken to not unreasonably restrict patients’ access to trials through their physicians by prohibiting physician referrals. When conflicts of interest are unavoidable, it is preferable to instead mitigate the negative effects through, for example, disclosure of conflicts of interest, as required by the CIOMS *International Ethical Guidelines* and the *Declaration of Helsinki* [40, 41]. In addition, efforts need to be made to ensure patients that declining to participate will neither impact their care nor their relationship with the physician.

## Is it ethical to leverage cognitive biases?

Historically, the literature on the ethics of informed consent has tended to assume that a valid consent must be the result of a deliberative decision-making process. However, more recent work in the psychology of decision-making has revealed that most decisions, including informed consent, are influenced by an array of deliberative and non-deliberative mechanisms, including a variety of factors that relate to the way in which the information is presented and framed [12, 42–45]. A recent study by Blaga, Frăţilă, and Meghea reported the effectiveness of leveraging non-deliberative behavioral influences to increase recruitment to a randomized controlled trial evaluating interventions to help pregnant women quit smoking [46]. One strategy was deployed in a Facebook ad for the trial that reads as follows: “Over 2000 future moms have already visited our website to learn more about the Quit Together program. Why not find out more about our free smoking cessation program?” [46]. This statement is intended to influence behavior by appealing to social norms. Because in many circumstances the actions of others can be a useful cue to what action is appropriate, people have the tendency to follow the behavior of others [47]. By appealing to the choice made by thousands of future mothers, the reader is encouraged to contemplate what constitutes socially appropriate behavior, which, consequently, makes it more likely that future mothers reading the ad will visit the website.

A second strategy was deployed on the trial’s website. Here, pregnant women were cautioned, “By not enrolling, you may miss out on 8 free counseling sessions with a counselor specialized in [motivational techniques]” [46, 48]. This behavioral influence works by exploiting “loss aversion,” which refers to the cognitive bias that people find losing something they already have more unpleasant than not gaining something of similar value [49]. As a result, framing information or consequences in terms of losses will tend to affect people’s behavior more strongly than framing in terms of gains. Thus, rather than describing free counseling sessions as a potential gain, framing them as a loss (i.e., “by not enrolling you may miss…”) makes it more likely that future mothers will choose to enroll.

Together with other influences that target non-deliberative decision-making, these influences resulted in a nearly threefold increase in this trial’s enrollment. But is it ethical to exploit cognitive biases to promote trial participation? On the one hand, the trialists in this case could be described as merely presenting true information about their study—and just like physician recommendations, this is already a part of routine practice for trial recruitment. On the other hand, we could describe the trialists as framing the information in a theoretically informed way that is expressly intended to manipulate the reader’s preferences. The trialists intend that this manipulation will lead to influencing the reader’s behavior and getting them to enroll in the trial.

This latter description does seem to raise ethical red flags. Such use of behavioral influences seems, prima facie, to undermine the decision-making autonomy of the potential participant [50–54]. The practice of obtaining informed consent, which derives from efforts to protect and enhance personal autonomy, is a key ethical protection in research. The provision of informed consent requires explicit, active deliberation and the evaluation of alternative options [24, 54]. Thus, insofar as a behavioral influence bypasses deliberation or seems to exert too much influence over the decision, it may seem to be unethical.

However, the assumption that valid, autonomy-respecting decision-making must be driven exclusively (or largely) by deliberative processes is questionable. For example, McKenzie and colleagues argue that unconscious behavioral influences can convey relevant information that can be highly valuable to decision-makers [55]. Christman has also offered a theory of autonomy that allows for actions to be authentic and, therefore, autonomous, even when they are influenced by the behavior of others [56, 57]. Within this theory, the authenticity of the decision to participate would not be undermined by behavioral influences provided that the participant would not reject the decision if they were to reflect critically on the process that led to that decision [56, 57]. The authenticity of a participant’s decision has also been identified as one of the core outcomes on which interventions on informed consent should be evaluated [58]. In addition, the observation that many (or most, or all) of our decisions are affected in some way by such non-deliberative influences of one kind or another, and it would seem that we cannot have a theory of autonomy, and conditions of valid informed consent, that always requires pure, deliberative decision-making. Decisions about trial participation will always be the result of some blend of active deliberation and non-deliberative mechanisms. Therefore, it would be overhasty to conclude that changing patient preferences in favor of trial participation from behavioral influence strategies necessarily conflict with autonomy.

So how then do we distinguish between autonomy-respecting and autonomy-undermining behavioral influences? Again, comprehensive protections are beyond the scope of this paper, but the ethics of minimal risk research may provide a helpful model. When evaluating whether a research procedure conducted within a trial constitutes minimal risk, a “daily-life standard” is often employed—that is, a risk is considered minimal for someone if the probability and magnitude of the anticipated harm are not greater than what they would encounter in daily life or in the course of routine care. Adapting this model for behavioral influences, we would argue that behavioral influence strategies could be considered prima facie autonomy-respecting if it is comparable to other uncontroversial behavioral influence strategies to which an individual is routinely exposed in their daily life. Although some considerations, for example, the involvement of vulnerable participants, could override this principle, we believe that ethics committees should start from the presumption that uncontroversial behavioral influences to which people are routinely exposed in daily life such as leveraging social norms and loss aversion in advertisements may be ethically acceptable. Before employing this rule, more work needs to be done to assess to which behavioral influences people are exposed in daily life, which of these may reasonably be regarded as uncontroversial, and why we ought to regard their use in research as permissible.

## Conclusion

The difficulty of recruiting enough research participants is a problem for many trials and for the health system in general. The examples discussed in the preceding sections highlight the promise and the perils of using behavioral influences to enhance trial recruitment. We have discussed just two examples of the 93 types of possible influences on behavior [23]. However, the unregulated use of behavioral influences presents serious risks to the integrity of the research and healthcare enterprise, and therefore, guidance for their ethical use is of critical importance. This is all the more pressing since behavioral influences are already used during trial recruitment [15], even though they are seldom mentioned explicitly in documents submitted to research ethics committees [15, 44]. Hence, the value of shifting to a richer, theoretical model for understanding behavior that can illuminate “how” and “why” particular recruitment practices may be effective or ineffective. Without this lens, behavioral influences will be largely invisible to ethical analysis. But with this theoretical understanding, we can begin to distinguish between ethically permissible and impermissible strategies and practices.

The existing evaluations of behavior change strategies in research recruitment only involve general discussions of a few types of behavioral influences, resulting in little guidance for trialists and research ethics committees for the many different types of behavioral influences [59, 60]. Therefore, we believe additional resources and guidance are needed. We have proposed here a few components of this guidance, but recognize that much more work is needed. In particular, we believe that trialists and ethics committees alike will need additional educational resources in order to better understand, detect, and ethically deploy behavioral influence strategies that may help to promote trial participation.

Finally, we believe that the potential harms and wrongs from behavior influences will require greater study. Whenever such strategies are employed, we would argue that trialists have an obligation to collect data on the experience of participants. Special attention needs to be given to collect and consider the experiences of participants from vulnerable and underserved groups (such as people with a low income, people with disabilities, children, minority sexualities, or ethnic or racial minorities), as it cannot be assumed that they have the same attitudes and responses to the use of behavioral influences. For example, indigenous participants place trust more reflectively and not in the same institutions as non-indigenous participants because of negative experiences with research in the past and might, therefore, respond differently to trialists that leverage use trust relations [31, 32]. Therefore, it is important that trialists collect the experiences of both vulnerable and less vulnerable (potential) participants and use those data to evaluate the influences being used.

A useful framework is the core outcome set for evaluating interventions on the informed consent process in clinical trials [58]. A “core outcome set” is a standardized list of outcomes that should be measured in all trials that investigate the same topic [58, 61]. As mentioned above, one of the core outcomes for informed consent intervention is the authenticity of a participant’s decision. Some of the other outcomes in this set include the comfort of a participant with the decision, empowerment, potential feelings of altruism, and the attrition from a trial. Some of these core outcomes, such as *comfort* and *attrition*, relate to our proposal that patient experience is relevant and essential for designing ethical recruitment strategies and could also be used to assess the interventions in the recruitment process [58]. Similarly, we believe that regret around a decision can be a powerful indicator of a lack of autonomy, and it is therefore incumbent on trialists to ensure (as much as possible) that their research participants do not regret their decision to enroll. Although the trial experience of participants has received only limited attention in the literature, there is some indication that a positive experience (especially a positive relationship with research staff) contributes to the participant’s willingness to remain in a trial and to participate again in the future [11, 62]. If this is indeed the case, a focus on participant experience in one trial could positively impact retention and recruitment in future trials [14]. Consequently, participants’ experience in the recruitment process and in the conduct of research will be critical to informing proper ethical guidance for the use of behavior influences.

### Supplementary Information

**Additional file 1.** Supplementary information.

**Additional file 2.**

## Data Availability

The data set for the analysis in Additional file 1 is available.
