# Supplementary material for: Changing patient preferences toward better trial recruitment: an ethical analysis
Source: Trials. 2023 Mar 28;24:233. doi: 10.1186/s13063-023-07258-4 (PMC10044713; doi:10.1186/s13063-023-07258-4)

Estimating the number of trials that terminate due to recruitment

# Methods

On August 15, 2022, we search Prism.Bio’s ClinicalTrials.gov API for all trials with a status of “Terminated” and trial start dates between January 1, 2010 and August 15, 2022. This search returned 13,335 records.

For each record, we process the text of the “why_stopped” field and classified the reasoning as related to recruitment if that text included any of the following strings: “recruit”, “accru”, “enrol”, “inclusion”, “not enough pa”. Note that many of these strings are truncated in order to capture variations on terms, such as “recruits”, “recruitment”, “accrue”, “accrual”, etc.

This specific list of terms was generated by investigating the raw “why_stopped” data and looking for patterns of words or phrases that often occurred and clearly indicated recruitment failures.

# Results

Based on this automated classification, we found 5,124 trials that terminated for reasons related to recruitment. This is 38.4% of all terminated trials.

We have included the complete data set of 13,335 trials along with this document as part of supplementary materials.

In Figure S1 below, we graph this data by trial start year. This shows how remarkably stable has been the proportion of trials that fail for reasons of recruitment.


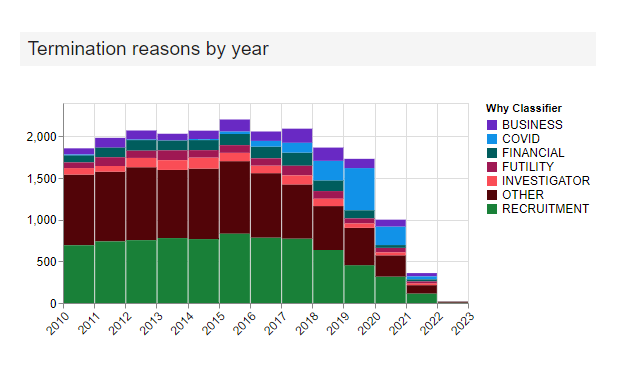

Supplement: Supplementary file 2 — Additional file 2. [file 13063_2023_7258_MOESM2_ESM.docx]
